# A meiotic driver alters sperm form and function in house mice: a possible example of spite

**DOI:** 10.1007/s10577-022-09695-4

**Published:** 2022-06-01

**Authors:** Lennart Winkler, Anna K. Lindholm

**Affiliations:** 1 Department of Evolutionary Biology, Bielefeld University, Konsequenz 45, 33615 Bielefeld, Germany; 2 Applied Zoology, TU Dresden, Zellescher Weg 20b, 01062 Dresden, Germany; 3 Department of Evolutionary Biology and Environmental Studies, University of Zurich, Winterthurerstrasse 190, 8057 Zurich, Switzerland

**Keywords:** *t* complex, gene drive, selfish genetic element, transmission ratio distortion, sperm motility, competition, social evolution

## Abstract

The ability to subvert independent assortment of chromosomes is found in many meiotic drivers, such as the *t* haplotype in house mice *Mus musculus*, in which the *t*-bearing chromosomal homolog is preferentially transmitted to offspring. This is explained by a poison-antidote system, in which developing + and *t* sperm in testes of + /*t* males are exposed to ‘poison’ coded by *t* loci, from which *t* sperm are protected, allowing *t* sperm an overwhelming fertilisation advantage in monogamous matings. This system is thought to result in poorly and normally motile sperm subpopulations within + /*t* sperm, leaving *t* sperm unharmed. Conversely, we found that the fastest quartile of sperm from + /*t* males swam more slowly, both forwards and along their travel path, and had reduced straightness and linearity, compared to the fastest quartile of + / + sperm. Moreover, sperm from + /*t* males had shorter tails and narrower heads than + / + sperm, and these morphological differences covaried with motility differences. Finally, + /*t* traits did not show evidence of bimodal distributions. We conclude that the *t* haplotype drive results in lasting damage to the motility of both + and *t* developing sperm, although previous studies indicate that + must be more harmed than *t* sperm. This damage to all sperm may explain the low success of + /*t* males in sperm competition with + / + males, seen in earlier studies. We propose that the harm the *t* causes to itself could be termed ‘spiteful’, which may also be common to other gamete-harming meiotic drive systems.

## Introduction

Independent assortment of chromosomes is the rule in diploids. However, the ability to manipulate transmission of one homolog over the other would be a great advantage to loci on the transmitted homolog, as they would increase in frequency. This ability is found in many loci that cause meiotic drive. Meiotic drive includes processes such as preferential transmission in females of driver-carrying chromosomes into ova rather than polar bodies, and gamete-killing in males that acts during spermatogenesis to damage or destroy rival gametes that inherit the alternative (wildtype ( +)) homologous chromosome, instead of the driving chromosome (Sandler and Novitski 1957; Burt and Trivers 2006; Zanders and Unckless 2019). A consequence of drive is the reduction in transmission of the + homologous chromosome to the next generation. Fitness of the offspring inheriting the driving homolog may also be reduced, as drivers and loci linked to them often harm organismal function (Zanders and Unckless 2019). These harms select for the evolution of loci that block drivers, by rendering them ineffective, or by behavioural or ecological mechanisms that allow individuals to avoid mating with driver carriers (Price et al. 2020). This in turn selects for drivers to maintain or increase their effectiveness, in an intra-genomic arms race, which may lead to multiple adaptations of the driver to maintain or enhance its advantage (e.g. Muirhead and Presgraves 2021).

One mechanism of drive that results in preferential transmission of driver-bearing chromosomes is the poison-antidote system (Willison 1999; Burt and Trivers 2006; Bravo Núñez et al. 2018). In individuals heterozygous for such a driver, developing + gametes are harmed or killed. This system requires two products that are controlled by the driver: a poison that spreads through shared cytoplasm to affect + and driver-bearing gametes, and an antidote that specifically rescues driver-bearing gametes from the poison.

One of the best understood poison-antidote systems is the *t* haplotype in house mice *Mus musculus*. It consists of at least four driver loci and a *cis*-active rescue locus (Herrmann et al. 1999; Bauer et al. 2005, 2007, 2012; Charron et al. 2019) bound together within linked chromosomal inversions, about 40 Mb in size (Kelemen and Vicoso 2018; Lindholm et al. 2019). The rescue locus is a *t*-specific allele of the gene *Smok*, a sperm motility kinase (Herrmann et al. 1999). The driver loci are involved in signalling pathways that regulate expression of *Smok*, and their activity results in hyperactivation of the + Smok allele, but not of its *t* allele (Herrmann et al. 1999; Amaral and Herrmann 2021). As *Smok* is thought to be upstream of genes affecting sperm motility, dysregulation of *Smok* in + spermatids leads to poor motility of + sperm (Amaral and Herrmann 2021). The result is up to 100% transmission of the *t* haplotype to offspring in crosses of + /*t* males with + / + females, although transmission rates vary by *t* haplotype variant (Silver 1985; Lindholm et al. 2013; Manser et al. 2017). At least 16 *t* haplotype variants are known (Klein et al. 1984).

Sperm of + */t* males have lower mean velocity and lower mean straight line (also called progressive) motility compared to + / + males (Olds-Clarke and Johnson 1993; Sutter and Lindholm 2016; Amaral and Herrmann 2021). This has been linked variously to abnormal flagellar function (Olds-Clarke and Johnson 1993), premature hyperactivation (Olds-Clarke 1989) and a premature acrosome reaction (Brown et al. 1989). Hyperactivation leads to changes in motility that enable sperm to reach and fertilise the egg (Ho and Suarez 2001), with hyperactivated sperm swimming less straight than activated sperm. The acrosome reaction releases enzymes necessary to penetrate the zona pellucida of the egg, making sperm capable of fertilisation (Jin et al. 2011). Impaired Rho small GTPase RAC1 signalling has also been linked to altered + /*t* sperm performance (Amaral and Herrmann 2021). These motility changes are thought to specifically affect + sperm, preventing them from reaching the egg (Olds-Clarke and Johnson 1993; Olds-Clarke 1997; Amaral and Herrmann 2021). Thus, two subpopulations of sperm are expected in sperm of + /*t* males: a slower and less progressive fraction, and a rescued, normally motile fraction (Amaral and Herrmann 2021).

Set against this expectation of poorly motile and normally motile sperm fractions in + /*t* males is evidence of low overall sperm quality in + /*t* males. When females mate with a + / + and a + /*t* male in the same oestrus cycle (in random order), a paternity share for the + /*t* male of one-third is expected if 50% of sperm from + */t* are uncompetitive (Sutter and Lindholm 2015). However, empirical data indicate + /*t* paternity shares in different *t* haplotype variants of 0.13 (Sutter and Lindholm 2015) and 0.24 (Manser et al. 2017). This suggests instead an overall sperm quality disadvantage also affecting *t* sperm, with similar effects seen in other sperm-killing meiotic drive systems (Price and Wedell 2008; Zanders and Unckless 2019; Verspoor et al. 2020).

In this study, we test the prediction from the poison-antidote hypothesis (Bravo Núñez et al. 2018; Amaral and Herrmann 2021) of two sperm subpopulations from + /t males—fast and slow, corresponding to a highly motile, rescued *t* fraction, and a poorly motile + fraction. In + */t* males, 50% of spermatids carry the *t* allele and 50% the + allele (Hammerberg and Klein 1975), but unfortunately, there is no method to directly label sperm to genotype to cleanly compare performance. However, following from Amaral and Herrmann (2021), we expect the top quartile (top 25%) of forwards moving sperm within a sample of + /*t* sperm to consist mainly of *t* sperm. This fast fraction is also likely to be more viable (Mossman et al. 2009) and more successful in fertilisation (Smith et al. 1989; Gomendio and Roldan 2008; Simmons and Fitzpatrick 2012). We therefore compare the motility of the top quartiles of sperm between + */t* and + / + males, based on the measure of straight line velocity, quantifying the effect of the *t* haplotype, while accounting for variation between individuals. We also test for differences between sperm from + / + and + /*t* males in morphological traits (head length and width, midpiece length and flagellum length), as these can influence motility (Fitzpatrick and Lüpold 2014), and a physiological trait (acrosome reaction status) previously suggested to be influenced by *t* (Brown et al. 1989). Finally, we look for evidence of bimodal distributions of sperm traits that could represent damaged and rescued sperm subpopulations.

## Methods

### Study animals

House mice *Mus musculus domesticus* for this study were part of the experiment of Runge and Lindholm (2021) which tested for differences in dispersal between + / + and + */t* house mice placed into 7 m^2^ outdoor enclosures at the University of Zürich. The genotype *t/t* could not be included, as mice of this genotype die prenatally (Lindholm et al. 2013; Sutter and Lindholm 2015). Each enclosure included four nestboxes, four feeding sites and four drinking sites, as well as plastic walls, tubes, bricks, sticks, rocks and tiles to make the habitat more complex and to provision hiding places. Male and female house mice at equal sex and genotype ratios entered the enclosures at approximately 36 days of age, and were allowed to freely reproduce, for an average of 107 days (Runge and Lindholm 2021). In this study, we examined sperm characteristics of sexually mature males born in eight enclosures in 2017. More than 99% of offspring born in the enclosures did not use the option to disperse (Runge and Lindholm 2021), and dispersers were not included in the present study. The mice entering enclosures were bred in the laboratory from 34 breeding pairs but were derived from a long-term house mouse population study, founded by locally wild-caught house mice which by chance included + */t* individuals (Manser et al. 2011; König and Lindholm 2012; Lindholm et al. 2013). See Runge and Lindholm (2021) for full details. This *t* variant causes 90% drive (Lindholm et al. 2013). Genotype was determined by amplification of the *Hba-ps4* locus (Schimenti and Hammer 1990) following our usual procedures (Lindholm et al. 2013).

### Sperm motility analysis

When ending an enclosure experiment, all animals were captured. Males were euthanised with CO_2_ and dissected immediately afterwards. Sample sizes were 34 + /*t* and 46 + / + males. The *cauda epididymis* was removed and transferred to a pre-warmed (37 °C) petri dish containing a 1-ml bubble of modified human tubal fluid (mHTF) medium (*Irvine Scientific*, Catalog ID: 90,126, containing sodium chloride, potassium chloride, magnesium sulphate, potassium phosphate, calcium chloride, sodium bicarbonate, HEPES, glucose, sodium pyruvate, sodium lactate, gentamicin and phenol red) with 5 mg/ml of bovine serum albumin (BSA; Sigma-Aldrich) covered with mineral oil. The mHTF medium does not require the use of a CO_2_ incubator. The epididymis was then cut open with three cuts, the epididymis was removed after 10 min, and the dish was incubated at 37 °C for 2 h. After 2 h of incubation, sperm is thought to have started capacitating (Larson and Miller 1999) and differences in this process can be measured. One 4 µl sample from the centre of the bubble was taken with a pipette and transferred to a pre-warmed (37 °C) Leja4 slide (20 µ). Samples with high sperm concentration (estimated by the amount of ‘shading’ of the mHTF medium) were diluted 1:1 with 37 °C warm mHTF medium prior to the transfer to the slide.

The Leja4 slide was then placed on a heating stage (37 °C) under an Olympus CX41 phase-contrast microscope and the sperm were analysed at 40 × magnification with the *MouseTraxx* (Mouse Traxx, Hamilton Thorne, Beverly, MA, USA) program using the standard analysis setup for mice*.* Specifically, sperm tracks were captured by recording 30 frames at a 60 Hz with minimum contrast set to 35 and minimum cell size at 5 pixels. The analysis of the MouseTraxx CASA system includes sperm concentration and parameters of velocity (average-path and straight line velocities, track speed, lateral amplitude and beat frequency of the flagellum, straightness and linearity). Average-path velocity (VAP) is defined as the smoothed average sperm track velocity, while straight line velocity (VSL), also called progressive velocity, captures the movement in a straight line. Track speed, also called curvilinear velocity (VCL), captures the velocity of sperm using every single frame. Straightness is defined as VSL/VAP*100 and therefore captures the percentage of forward movement relative to the path velocity. Finally, linearity is defined as VSL/VCL*100 and represents the deviation of the sperm movement from a straight line based on single frames. Straight line velocity is considered an especially good measure of sperm velocity, as in mammals mean VSL correlates with both maximum VSL, as well as with male fertility (Moore and Akhondi 1996; Gomendio and Roldan 2008). The CASA velocity results were only used for analysis if at least 200 sperm were counted and 50 of those were classified as rapid.

### Sperm morphology

We investigated sperm morphology by measuring head, midpiece and tail lengths by hand in ‘ImageJ’ (Schneider et al. 2012) using the segmented line tool. The images were stained with Coomassie brilliant blue (CBB) (as in the acrosome analysis). Lengths were calibrated using a pictured micrometre (Neubauer/Scherf Präzision). We measured head length from the base of the head to the tip and head width at the widest part of the head (both excluding the sperm hook). The same calibration factor was used throughout all measurements. We measured a total of 1000 sperm from 50 individuals, 25 + /*t* and 25 + / + . In addition, we re-measured 100 sperm a few weeks after the first measurement, to estimate measurement repeatability. These measurements were taken from a random subsample of 31 different individuals. Repeatability was high for all measurements, with the lowest intra-class coefficient (ICC) of 0.74 for midpiece length (see Table S1). In addition to the direct measures of length and width, we added the head length-to-width ratio as a measure of head shape and the head length to flagellum length (including midpiece and tail) ratio.

### Acrosome staining

The staining methods are a modified version of the staining protocol to determine the acrosomal status of sperm suggested by Larson and Miller (1999). We validated our scoring in a pilot study by comparing untreated samples with samples challenged with calcium ionophore to induce acrosome reaction as in Larson and Miller (1999). Treatment of Ca^2+^ dissolved in DMSO left 9.5% of samples with intact acrosomes, while the DMSO control left significantly more acrosome-intact sperm (mean = 37.8%, *n* = 13, *p* < 0.0001), which were the expected results.

After 2 h of incubation, a 20 µl sperm sample from the centre of the HTF bubble was smeared on a slide and air dried. The slides were stained using freshly made CBB stain (40 ml H_2_O, 50 ml methanol, 10 ml acetic acid and 220 mg Coomassie blue) by incubating in a bath of CBB for 4 min and careful rinsing afterwards.

**Fig. 1:**
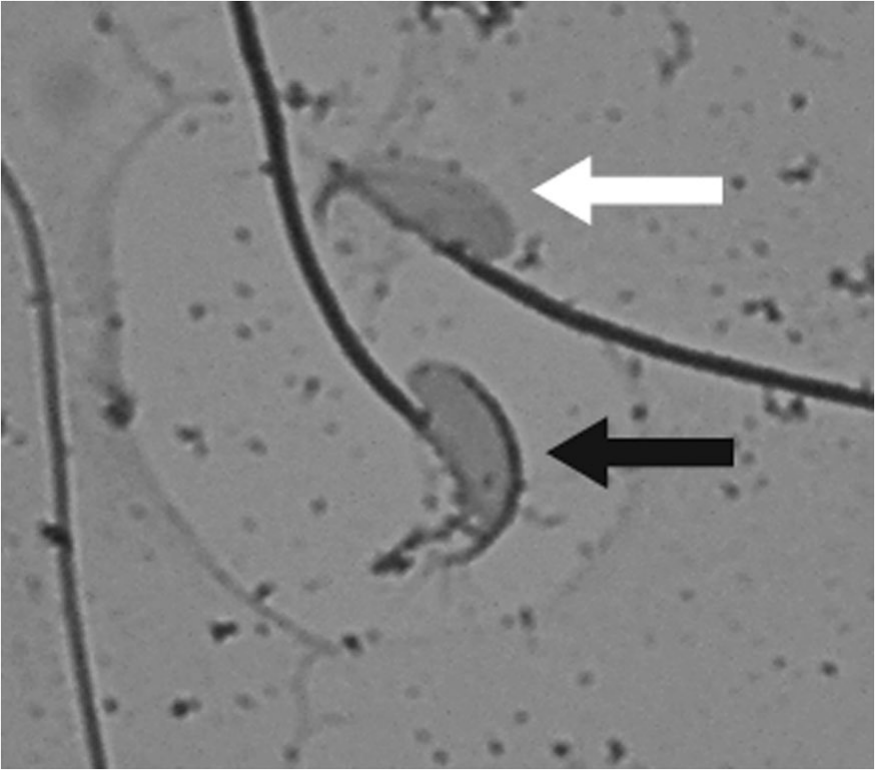
Greyscale microscope image of CBB stained sperm at 1000 × magnification. Black arrow indicates acrosome-intact and white arrow acrosome-reacted sperm

After slides were air dried (min. 4 h), samples were sealed with DPX mountant for histology (SIGMA) and 24 × 60 mm cover slips. Afterwards, pictures were taken at a magnification of 400 × with a Leica EC4 using the LAS EZ program (50% brightness, 0,7 gamma and 105 saturation) (see Fig. 1). At least 100 and a mean of 229 sperm were pictured for each sample. The presence or absence of the acrosome was determined by eye (see Fig. 1). Acrosome reaction scoring was highly repeatable over nine separate samples (*r*^2^ = 0.902).

### Statistical analyses

All statistical models were analysed using ‘R’ version V4.0.2 and ‘RStudio’ (RStudio Team 2015; R Core Team 2018). Generalised linear mixed models used package lme4 (Bates et al. 2015), with *p* values fit by REML. The effect of genotype was estimated in each model, with + */t* as the reference level. Animal ID was included as a random factor when there were repeated measures per male. Enclosure ID was explored as a second random factor, but returned variance estimates of zero, and was then dropped.

For sperm motility comparisons, we analysed two subsets of the sperm motility data in addition to the full data. First, we restricted the data per male to sperm with a straight line velocity in the top 25% of the sample. Second, we restricted the dataset further. Under the poison-antidote hypothesis, 50% of sperm of a + /*t* male will be damaged in their motility. Therefore, any differences in sperm competition success between + /*t* and + / + males could be due to + / + males having a 50% larger number of functional sperm. To account for this advantage in sperm numbers in + / + males, we randomly removed half of their motility data from this analysis. Any remaining differences between + / + and + /*t* males’ sperm should thus be due to differences in the competitive fraction of sperm and not only due to differences in numbers.

For analyses of the acrosome reaction, the response variable was the proportion of sperm that had undergone the acrosome reaction. As models were over-dispersed, the data was fitted using a quasibinomial model.

Outliers of more than 10 times the standard deviation in sperm motility traits were excluded from the analyses (< 0.1% of data points per trait). In the sperm morphology analyses, sperm that deviated more than 3 times the standard deviation from the mean were removed as outliers. From midpiece length, tail length, head length and head width measures, respectively, 14, 16, 11 and 1 outliers were removed. Removed outliers did not qualitatively alter the results.

To assess bimodality of the distributions of traits measured in + / + and + /*t* males, we compared coefficients of variation (CV), because bimodality should lead to increased within-individual variation. Nevertheless, increased CVs are not a conclusive test for bimodality, as this increase can have multiple reasons stemming from different data distributions. Hence, to further test for bimodality, we used the R package ‘mode’ (Deevi and 4D Strategies 2016) to estimate how close to a bimodal distribution each of the datasets were. We therefore calculated the bimodality coefficient that estimates on a scale from 0 to 1 the proximity to bimodality. In addition to this measure of bimodality, we used the ‘Hartigan’s dip statistic’ (HDS) (Hartigan and Hartigan 1985; Maechler 2016) as an alternative statistical approach (Freeman and Dale 2013). We performed all analyses concerning bimodality using the full data (i.e. not the subset data).

## Results

###  + */t males have reduced sperm motility compared to* + */* + 

**Fig. 2:**
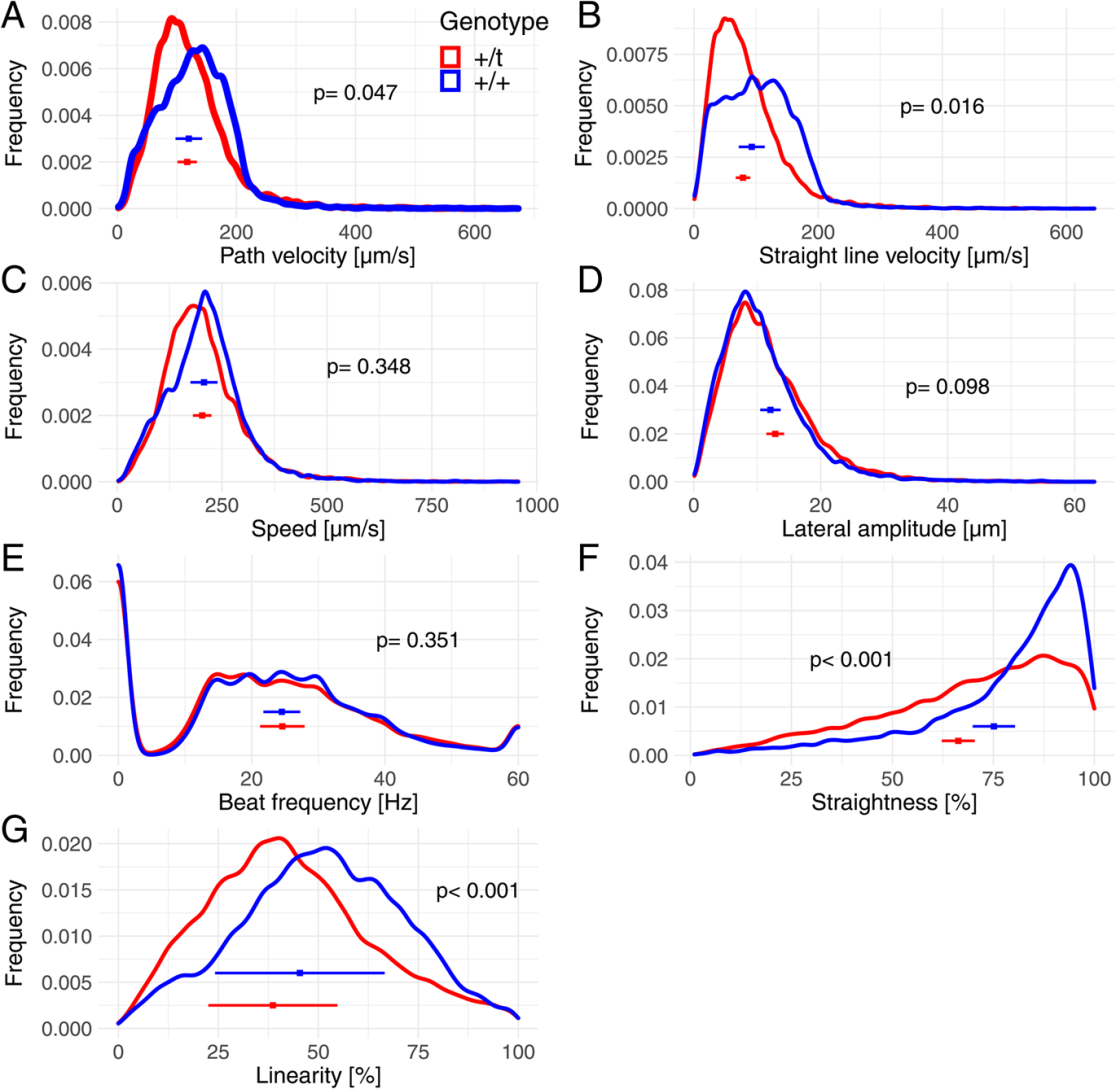
Density plots of individual sperm parameters as measured by CASA in red + /t (*n* = 34 males) and in blue + / + (*n* = 46 males), including means and standard deviations for mean trait of individuals. Statistical analysis results are in Table S2

First, we analysed differences in sperm motility parameters of + /*t* and + / + sperm samples using the full data (Fig. 2; see Table S2 for model summary). Sperm from + /*t* males had a significantly lower path and straight line velocity compared to sperm of wildtype males (Fig. 2A and B; see Table S2 for model summary). In addition, sperm of + /*t* males were significantly less straight and linear in their movement compared to sperm of + / + males (Fig. 2G and F). In contrast, there were no differences in speed, lateral amplitude or beat frequency of sperm between + /*t* and + / + samples (Fig. 2C–E).

We estimated using analysis of variance that genotype accounted for 5–8% of variance in sperm performance for straight line velocity, straightness and linearity, with estimates not significantly different from zero for other measures (Table 1). In comparison, individual differences accounted for 5–12% of variance.

**Table 1:** Analysis of variance (fit by REML) for individual sperm tracks and sperm morphologies (*n* = 200 sperm per male). Shown per trait are variance components in % of genotype, individual and residual, and ANOVA *p* values (via ML) for difference in models if genotype, or individual, is dropped

Variable	Variance attributed in %				
	Genotype	*p* value	Individual	*p* value	Residual
Path velocity (µm/s)	0.98	0.363	11.95	< 0.001	87.07
Straight line velocity (µm/s)	4.75	< 0.001	11.23	< 0.001	84.02
Speed (µm/s)	0.15	0.999	8.33	< 0.001	91.52
Lateral amplitude (µm)	0.31	0.556	5.23	< 0.001	94.46
Beat frequency (Hz)	0.01	0.999	5.39	< 0.001	94.59
Straightness (%)	8.11	< 0.001	6.15	< 0.001	85.74
Linearity (%)	6.30	< 0.001	7.86	< 0.001	85.84
Midpiece length (µm)	0.00	1.000	25.87	< 0.001	74.12
Tail length (µm)	22.45	< 0.001	23.94	< 0.001	53.61
Head length (µm)	0.42	1.000	15.82	< 0.001	83.76
Head width (µm)	3.70	0.350	21.89	< 0.001	74.41
Head shape ratio	8.12	0.023	15.57	< 0.001	76.31
Head–tail ratio	7.50	0.029	15.13	< 0.001	77.37

###  + */t males sperm motility remains lower in subset of high motility sperm*

**Fig. 3:**
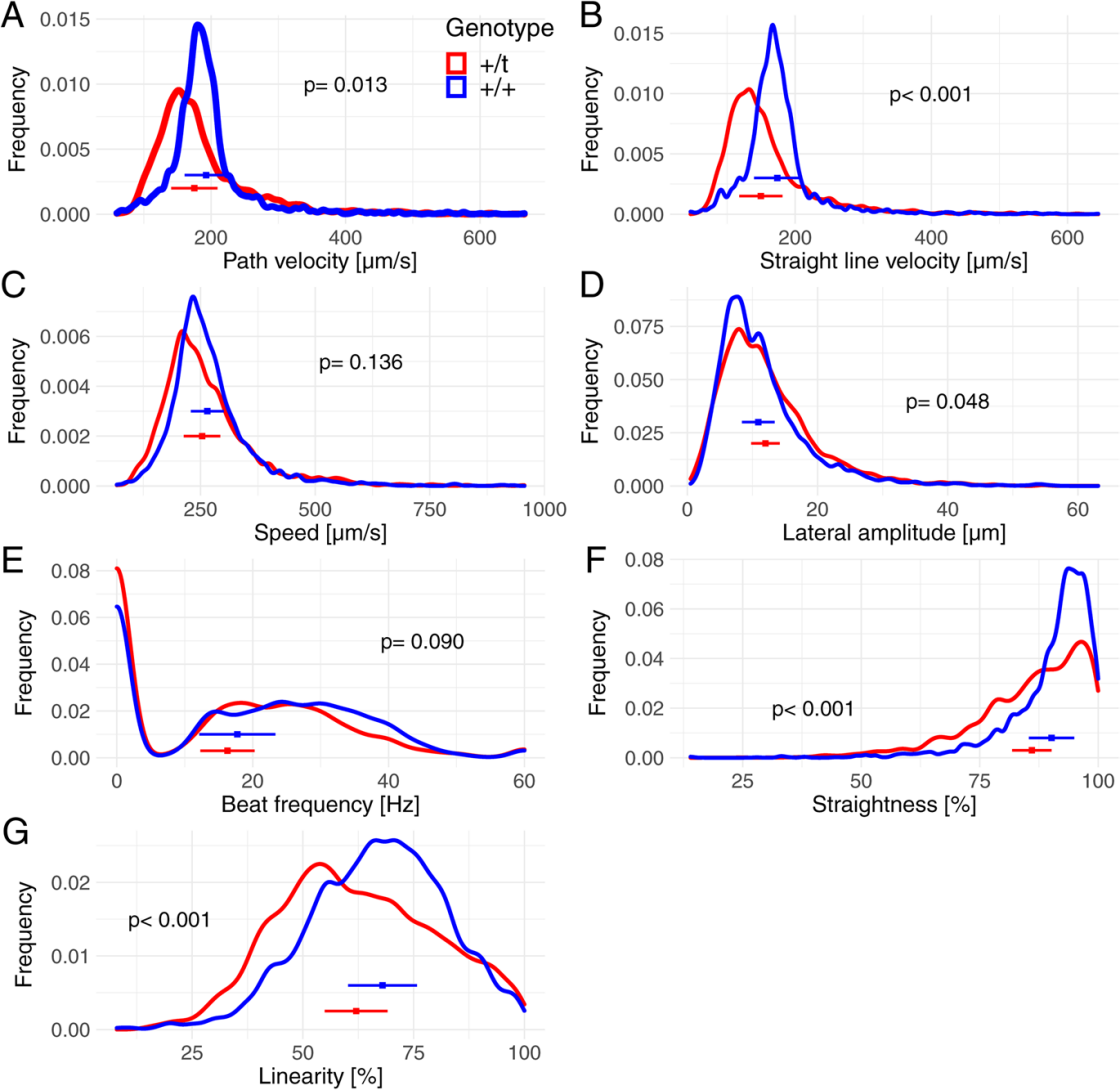
Density plots of individual sperm parameters as measured by CASA in red + /*t* (*n* = 34 males) and in blue + / + (*n* = 46 males) only including the 25% fastest sperm per sample, including means and standard deviations for mean trait of individuals. Statistical analysis results are in Table S3

Next, we subset the data to contain only sperm with a straight line velocity in the top 25% of the sample. The top quartile of sperm of + /*t* males had lower path and straight line velocities and swam less straight and less linearly compared to the top quartile sperm of + / + males (Fig. 3; see Table S3 for model summary). In addition, there was a marginally significant difference in lateral amplitude between sperm of + / + and + /*t* males. No differences were found in track speed and beat frequency. Over the entire sample, sperm concentration was not different between the genotypes (two-sided Welch two sample *t* test: *t* = 0.99; df = 78; *p* value = 0.331). We also controlled for potential differences in the sample pool compared: if we expect 50% intact sperm in + */t* samples but 100% intact sperm in + / + samples, then differences due to a halved sample pool for + */t* males are a possible explanation for finding reduced performance. In a next step, we randomly removed 50% of sperm per + / + sample and compared the remaining sample to the top quartile + */t* sperm. Results remained similar to the previous analysis (Table S4).

Again, we estimated the variance that genotype accounts using analysis of variance. We found that in the top 25% of the sample, genotype accounted for 5–6% of variance in sperm performance for straight line velocity, straightness and linearity, with estimates not significantly different from zero for other measures (Table S5). In comparison, individual differences accounted for 8–23% of variance.

###  + */t male sperm had a shorter tail and narrower head compared to* + */* + *sperm*

**Fig. 4:**
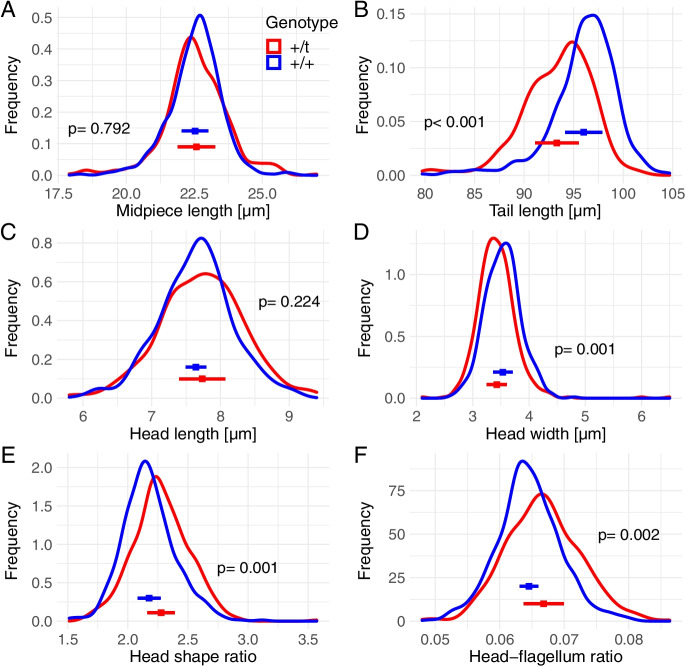
Density plots of sperm morphological traits for + /*t* males (red) and + / + (blue), including means and standard deviations for mean traits of individuals. **A** Midpiece length, **B** tail length, **C** head length, **D** head width, **E** head shape ratio (head length/head width) and **F** head-flagellum ratio (head length/(midpiece + tail length)). Statistical analysis results are in Table S6

We found a significant difference in mean tail length and head width, with sperm from + /*t* males having shorter sperm tails and a narrower head compared to + / + sperm (Fig. 4; see Table S6 for model summary). The mean midpiece length and head length of + /*t* and + / + males did not differ. We found both head shape (head length/head width) and the head-to-flagellum ratio (head length/(midpiece + tail length)) to be significantly influenced by genotype.

Increased sperm tail length covaried positively with straight line velocity, straightness and linearity (Figure S1 and Table S7), and head width with both path linearity and straightness (Figure S2 and Table S8) using the complete sperm motility data (i.e. not top 25%). However, there was no relationship between head shape or head-to-flagellum ratio and sperm motility parameters, apart from increasing head elongation predicting lower linearity and straightness (Figures S3 and S4 and Tables S9 and S10).

Genotype differences explained a significant amount of tail length variance (22%) but little to no variance for midpiece length, head length and head width (Table 1). Individual differences explained considerable variance for tail length (24%), head width (22%) and midpiece length (26%), while they explained less for head length (16%).

### No difference in acrosome reaction timing in + /t and + / + 

There was considerable variation between males in the percentage of acrosome-intact sperm found, ranging from a minimum of 31 to 86% acrosome-reacted sperm (Figure S5). However, we found no significant difference in the acrosomal status of + */t* and + / + males after 2 h of capacitation (estimate =  − 0.18, df = 83; std. error = 0.12, *t* value =  − 1.46, *p* value = 0.149; quasibinomial glm). The percentage of acrosome-intact sperm did not correlate with sperm velocity parameters (Table S11).

### *Evidence for bimodal distribution of* + */t sperm motility or morphology*

Plots of the distributions of trait values for + /*t* and + / + males (Figs. 2 and 3) show that the distributions might differ in modal values and might show increased variation in some traits. Nevertheless, motility and morphological traits of + /*t* sperm do not clearly exhibit bimodal distributions, as we demonstrate in the following analysis of the full data of sperm samples.

Using the coefficient of variation (CV), we tested if within-individual variation differed between sperm of + /*t* and + / + mice. We found significant differences in the CVs of straight line velocity, straightness and linearity between + /*t* and + / + , with higher CV for + /*t* in each case, but found no difference in other motility traits, or in sperm morphological traits (Table 2 and Figures S6 and S7).

**Table 2:** Comparison of coefficients of variation of sperm parameters for + / + and + /*t*. Comparison of means by two-sided Welch two sample *t* test allowing unequal variances

Variable	+ /*t*		+ / +				
	Mean CV	Sd	Mean CV	Sd	t	df	*p* value
Path velocity (µm/s)	50.53	10.19	49.03	14.04	0.58	85.85	0.565
Straight line velocity (µm/s)	66.46	11.25	59.10	20.47	2.15	79.14	0.034
Speed (µm/s)	46.08	11.37	44.68	11.82	0.56	81.32	0.577
Lateral amplitude (µm)	59.27	10.52	59.96	12.94	− 0.28	85.58	0.782
Beat frequency (Hz)	71.42	9.52	72.83	14.60	− 0.55	84.24	0.584
Straightness (%)	32.65	4.33	25.25	7.44	5.86	81.06	< 0.001
Linearity (%)	48.39	11.16	41.39	5.84	3.80	77.38	< 0.001
Midpiece length (µm)	4.23	1.56	3.57	1.21	1.67	45.32	0.101
Tail length (µm)	3.00	1.01	2.80	1.24	0.62	46.12	0.540
Head length (µm)	6.70	1.35	6.78	1.60	− 0.18	46.69	0.856
Head width (µm)	8.49	3.49	7.67	1.51	1.08	32.66	0.286
Head shape ratio	9.26	1.84	8.99	2.01	0.51	47.62	0.614
Head–tail ratio	7.29	1.65	7.19	1.74	0.20	47.87	0.840

We then tested sperm distributions using the bimodality coefficient, for which bimodality is considered at values larger than 0.56 (Pfister et al. 2013). We found for straight line velocity that sperm from + /*t* males had a higher bimodality coefficient than + / + males; however, the values were below the 0.56 threshold (0.42 and 0.25, respectively). The opposite was the case for straightness, with a larger bimodality coefficient for + / + compared to + /*t* (0.66 and 0.56, respectively). Finally, there was little difference in the bimodality coefficients of linearity with 0.39 for + / + and 0.43 for + /*t*.

We also used ‘Hartigan’s dip statistic’ (HDS) to estimate the modality of sperm velocity parameters, with a larger *D* value indicating a more ‘multimodal-like’ distribution (Freeman and Dale 2013). All tested parameters were significantly different from unimodality, but given the very large sample size (min. 68,500), this is not surprising. Hence, it is more reasonable to directly look at the test statistics. Here, we find that for straight line velocity, + / + males had a larger *D* statistic (*D* = 0.004) than + /*t* males (*D* = 0.003). For straightness, we find that + / + males again exhibited a larger *D* value compared to + /*t* males (*D* = 0.02 and 0.01, respectively), while *D* values were similar for the genotypes concerning linearity (*D* = 0.01).

## Discussion

The poison-antidote model (Bravo Núñez et al. 2018; Amaral and Herrmann 2021) predicts that sperm from + /*t* males consist of two subpopulations: poisoned and slow + sperm, and rescued and high-performing *t* sperm. We investigated if high-performing sperm from + /*t* males have a similar motility to sperm from + / + males, predicting no difference. We found that sperm from + /*t* males swam more slowly, both forwards and along their travel path, and had reduced straightness and linearity of their travel paths compared to + / + . While these are signs of hyperactivation (Neill and Olds-Clarke 1987), we did not find that the beat frequency of the flagellum was different between + / + and + /*t* males, contrasting with previously reported results (Olds-Clarke and Johnson 1993). The *t* haplotype accounted for up to 8% of variation between males in these sperm motility traits, less than that attributed to differences between individual males. Coefficients of variation in straight line velocity, straightness and linearity, but not other traits, were greater in the entire sperm sample of + /*t* compared to + / + , which is only partially consistent with predictions of two sperm subpopulations, and tests of bimodality did not provide evidence of subpopulations.

The strongest effect of the *t* haplotype seen in this study was its effect on sperm morphology. Sperm from + /*t* had shorter tails and narrower heads. Genotype differences accounted for 22% of tail length variation. In zebra finches (*Taeniopygia guttata*), a strong genetic effect on sperm tail length has also been detected, with sperm tail length phenotypically and genetically correlated with straight line sperm velocity (Mossman et al. 2009). A supergene within a Z chromosome inversion influences this sperm tail length variation (Kim et al. 2017). In our sample, straight line sperm velocity showed a positive phenotypic correlation with tail length, and path straightness correlated positively with head width. A previous study in house mice found that only sperm midpiece length predicted sperm velocity (Firman and Simmons 2010). The possible mechanistic bases of our results are unclear, but a longer tail is expected to provide more propulsive force, and a wider head may increase drag (Gomendio and Roldan 2008; Humphries et al. 2008), although empirical support is mixed (Simmons and Fitzpatrick 2012; Fitzpatrick and Lüpold 2014; Bennison et al. 2015).

The *t* haplotype is a supergene, containing several hundred genes linked together within chromosome inversions and inherited as a unit (Kelemen and Vicoso 2018; Lindholm et al. 2019). In haploid sperm cells, differences have been found between + and *t* sperm cells in gene expression (Cebra-Thomas et al. 1991; Veron et al. 2009). In testes from mice of the same strain as used in this study, there are differences between + /*t* and + / + in gene expression, particularly of spermatogenesis genes (Lindholm et al. 2019). Some of the expression differences found in Lindholm et al. (2019) map to genes whose products localise to sperm tails (Huw et al. 1995; Pilder 2012). There are therefore several candidates for sperm morphological differences.

We found that the acrosome reaction timing was not different between + /*t* and + / + males. While there is conflicting evidence (Brown et al. 1989), our results add to the evidence that there is no difference in acrosome reaction timing between + /*t* and + / + sperm (Olds-Clarke and Johnson 1993; Olds-Clarke 1997).

Our results provide evidence that the *t* haplotype results in lasting damage to both + and *t* developing sperm, harming in particular their forward movement. Our tests were conducted within a single context, but numerous studies have shown that sperm from + /*t* sperm on average have a worse swimming performance than sperm from + / + (Olds-Clarke 1983, 1997; Brown et al. 1989; Sutter and Lindholm 2016). Experiments using the oviductal environment in vivo also support this interpretation (Tessler and Olds-Clarke 1981). Furthermore, these results accord with experiments showing that + /*t* males are highly inferior sperm competitors (Sutter and Lindholm 2015; Manser et al. 2017). If all sperm of + /*t* males are somewhat affected in their motility by the meiotic drive mechanism, a fatal impact on sperm competitiveness seems likely. Furthermore, general differences in motility might contribute to an explanation of the puzzling result that + /*t* males never enjoy a first-male fertilisation advantage when two males mate with the same female, regardless of whether their competitor is + / + or + /*t* (Sutter and Lindholm 2016). In house mice, the first male to mate typically has a fertilisation advantage (Firman and Simmons 2008).

Damage to both + and *t* sperm by the driver loci of the *t* haplotype suggests that it harms both the opposing allele (+ sperm) and itself (*t* sperm). Nevertheless, the harm cannot be truly indiscriminate for drive to work. We hypothesise that + sperm are harmed more severely than *t* by incomplete compensation of harm. In this way, the poison-antidote system could still provide an evolutionary advantage to the *t* haplotype by increased transmission.

For harm to be an evolutionarily stable strategy (Hamilton 1964), it needs to have a feedback benefit to the actor. This is met by the enhanced transmission rate of the *t,* 90% for this *t* variant (Lindholm et al. 2013), giving the *t* a selfish benefit in local competition for fertilisation with + sperm from the same male. Looking at wider fitness effects, the impaired sperm competitiveness of + /*t* males, due to the harm of the *t* haplotype to all sperm, lowers the transmission rate of the *t*. This cost has been measured under controlled conditions as a 87% probability of failure for + /*t* to fertilise eggs (Sutter and Lindholm 2015). In our wild study population, the *t* went extinct in less than a decade (Runge and Lindholm 2018; Manser et al. 2020). In natural and experimental populations, sperm competition between males is detrimental to *t*’s fitness (Manser et al. 2011, 2017, 2020). A polyandry (female multiple mating) rate of 50–70% has been predicted to lead to local extinction of *t* (Manser et al. 2020). Thus, the *t* haplotype appears to have harmed its fitness under conditions of sperm competition with other males to gain a benefit under monogamous conditions. The *t* haplotype might therefore be termed ‘spiteful’ as spiteful acts reduce the fitness of both actor and recipient (Hamilton 1970; Gadagkar 1993; Foster et al. 2001; Vickery et al. 2003; Burt and Trivers 2006). The evolution of spite requires kin discrimination, and the direction of harm towards non-relatives (Patel et al. 2020). From the point of view of the *t*, + sperm are non-kin. Kin recognition is provided by a *t*-specific allele of the target of the four driver loci, the locus *Smok*, which shows haploid-specific expression (Herrmann et al. 1999; Veron et al. 2009). Whether the *t* might be considered spiteful, or selfish, depends on the costs and benefits it experiences, which vary with local conditions such as mating context and population + /*t* frequency.

Similar effects might be seen in other drive systems. In many meiotic drive systems, + sperm are killed during development and never appear in the ejaculate. This leads to up to 50% reduction in ejaculate size, impairing success in inter-ejaculate sperm competition, and limiting successful remating (reviewed in Verspoor et al. 2020). Stalk-eyed flies *Teleopsis dalmanni* carrying *SR* (*Sex Ratio* distorter) are an interesting exception as they appear to compensate for the sperm number reduction by increasing sperm production (Meade et al. 2018, 2020). Other systems appear more like *t*, but with a reduction in sperm quality for unknown reasons (Price and Wedell 2008). *Drosophila simulans*, for example, show lowered sperm viability (Angelard et al. 2008) and *D. pseudoobscura* impaired maintenance of sperm quality under long-term storage (Giraldo‐Perez et al. 2016).

Other drive systems have been considered possibly spiteful. One example occurs in the red flour beetle *Tribolium castaneum* (Wade and Beeman 1994), in which the driving *Medea* allele in *Tribolium* has been described as possibly spiteful, as it eliminates offspring that did not inherit a *Medea* copy (Frank 1995). Similarly, the endosymbiont *Wolbachia* causing cytoplasmic incompatibility has been described as spiteful (Gardner and West 2006). Nevertheless, convincing examples of spiteful relationships seem extremely rare.

## Conclusions

We propose a modification of the poison-antidote hypothesis so that the hypothesised rescue of *Smok* dysregulation in *t* spermatids is incomplete, all sperm are harmed, but + are harmed more than *t*. The fitness costs of this harm place the *t* in the selfish to spiteful spectrum. The cause of reduced sperm progressive motility is unclear, but altered sperm morphology may play a role. The *t* appears to be an exception to the conclusion that meiotic drivers do not distort organismal traits but supports the prediction that such distortion should be weak where it is found (Scott and West 2019). The reduced motility of + sperm in + /*t* ejaculates has previously been linked to excessive RAC1 signalling, with the experimental addition of a RAC1 inhibitor leading to increased linearity of sperm tracks (Amaral and Herrmann 2021). A next step would be to test effects of RAC1 signalling inhibition in the present system. The driver system is likely to be highly complex, and suboptimal compensation might be due to constraints in controlling spermatogenesis. Incomplete rescue is possibly the result of evolved suppression to the driver, if it increases transmission of + alleles. The evolution of four different *t* driver loci that act additively to cause a high rate of transmission distortion is suggestive of multiple cycles of adaptation by the driver to increase drive and counter-adaptation by the rest of the genome to restore Mendelian inheritance. It is unclear whether the effects on sperm described here are general to all *t* variants. It will also be interesting to see whether comparable effects on sperm morphology and motility will be found in other meiotic driver systems. While the evolution of true spitefulness should be an uncommon event, meiotic drivers like the *t* haplotype could provide a fruitful evolutionary context for this rarity.

## Supplementary Information

Below is the link to the electronic supplementary material.

Supplementary file1 (PDF 1.06 MB)

## Data Availability

Data are deposited  at the Dryad Digital Repository at 10.5061/dryad.x95x69pmc.
