# Supplementary material for: A meiotic driver alters sperm form and function in house mice: a possible example of spite
Source: Chromosome Res. 2022 Jun 1;30(2-3):151–64. doi: 10.1007/s10577-022-09695-4 (PMC9508062; doi:10.1007/s10577-022-09695-4)
Supplement: Supplementary file 1 — Supplementary file1 (PDF 1.06 MB) [file 10577_2022_9695_MOESM1_ESM.pdf]

Supplement for

A meiotic driver alters sperm form and function in house mice: a possible example of spite

Lennart Winkler and Anna K Lindholm

Table of contents

Table S1 ICCs of repeated sperm morphology measurements ... 2
Table S2 Linear mixed model of velocity measurements of individual sperm for +/*t* and +/+ ... 2
Table S3 Linear mixed model of velocity measurements (25% most progressive sperm fraction) ... 2
Table S4 Linear mixed model of velocity measurements (50% of +/+ males' sperm and the 25% of the most progressive sperm) ... 3
Table S5 Analysis of variance for individual sperm tracks (top 25%) ... 3
Table S6 Linear mixed model of sperm morphology measurements for +/*t* and +/+ ... 3
Table S7 Effect of tail length on velocity measurements ... 4
Table S8 Effect of head width on CASA velocity measurements using lm ... 4
Table S9 Effect of head shape on CASA velocity measurements using lm ... 4
Table S10 Effect of head-to-tail ratio on CASA velocity measurements using lm ... 5
Table S11 Effect of acrosome reaction status on CASA velocity measurements using lm ... 5
Fig. S1 Relationship between tail length and sperm motility (25% most progressive sperm fraction) ... 6
Fig. S2 Relationship between head width and sperm motility (25% most progressive sperm fraction) ... 7
Fig. S3 Relationship between head shape ratio and sperm motility (25% most progressive sperm fraction) ... 8
Fig. S4 Relationship between head-flagellum ratio and sperm motility (25% most progressive sperm fraction) ... 9
Fig. S5 Boxplot of +/*t* haplotype and +/+ males by percentage of intact acrosomes ... 10
Fig. S6 Boxplot of coefficients of variation of sperm velocity parameters for +/+ and +/*t* males ... 11
Fig. S7 Boxplot of coefficients of variation of sperm morphology parameters for +/+ and +/*t* males ... 12

Table S1 ICCs of repeated sperm morphology measurements. *n*=100.

Variable	ICC
Midpiece length [µm]	0.74
Tail length [µm]	0.74
Head length [µm]	0.82
Head width [µm]	0.81

Table S2 Linear mixed model (fit by REML) of CASA velocity measurements of individual sperm for +/*t* and +/+. Sample ID included as random effect.

Variable	Estimate [+/+]	Std. error	df	t value	p-value
Path velocity [µm/s]	13.69	6.80	79.79	2.01	0.047
Straight line velocity [µm/s]	16.40	6.68	79.76	2.45	0.016
Speed [µm/s]	7.89	8.36	80.23	0.94	0.348
Lateral amplitude [µm]	-0.56	0.33	82.14	-1.67	0.098
Beat frequency [Hz]	1.08	1.16	78.60	0.94	0.351
Straightness [%]	2.47	0.50	83.84	4.98	<0.001
Linearity [%]	5.06	1.32	82.67	3.84	<0.001

Table S3 Linear mixed model (fit by REML) of CASA velocity measurements only including sperm with 25% highest straight line velocity per sample. Sample ID included as random effect.

Variable	Estimate [+/+]	Std. error	df	t value	p-value
Path velocity [µm/s]	17.29	6.83	80.33	2.53	0.013
Straight line velocity [µm/s]	23.90	6.91	81.47	3.46	<0.001
Speed [µm/s]	11.66	7.74	80.56	1.51	0.136
Lateral amplitude [µm]	-0.95	0.47	76.20	-2.01	0.048
Beat frequency [Hz]	1.72	1.00	75.74	1.71	0.090
Straightness [%]	4.17	0.96	83.52	4.35	<0.001
Linearity [%]	5.84	1.57	82.00	3.73	<0.001

Table S4 Linear mixed model (fit by REML) of CASA velocity measurements only including 50% of +/+ sperm and +/*t* sperm with 25% highest straight line velocity per sample. Sample ID included as random effect.

Variable	Estimate [+/+]	Std. error	df	t value	p-value
Path velocity [µm/s]	10.24	4.82	84.36	2.12	0.036
Straight line velocity [µm/s]	19.59	4.56	83.74	4.30	<0.001
Speed [µm/s]	7.68	5.91	83.01	1.30	0.197
Lateral amplitude [µm]	-0.67	0.34	82.24	-1.96	0.054
Beat frequency [Hz]	-0.86	0.83	78.45	-1.03	0.304
Straightness [%]	8.85	1.20	82.14	7.36	<0.001
Linearity [%]	7.75	1.33	83.12	5.82	<0.001

Table S5 Analysis of variance (fit by REML) for individual sperm tracks (top 25%). Shown per trait are variance components in % of genotype, individual and residual, and ANOVA p-values (via ML) for difference in models if genotype, or individual, is dropped.

	Variance attributed in %				
Variable	Genotype	p-value	Individual	p-value	Residual
Path velocity [µm/s]	2.55	0.162	19.78	<0.001	77.67
Straight line velocity [µm/s]	5.87	0.016	22.51	<0.001	71.62
Speed [µm/s]	0.39	0.911	12.75	<0.001	86.85
Lateral amplitude [µm]	0.64	0.432	8.29	<0.001	91.07
Beat frequency [Hz]	0.39	0.704	8.05	<0.001	91.56
Straightness [%]	6.64	<0.001	15.39	<0.001	77.96
Linearity [%]	5.17	0.007	16.66	<0.001	78.17

Table S6 Linear mixed model (fit by REML) of sperm morphology measurements for +/*t* and +/+. Sample ID included as random effect.

Variable	Estimate	Std. error	df	t value	p-value
Midpiece length [µm]	-0.04	0.17	47.49	-0.26	0.792
Tail length [µm]	2.71	0.58	47.64	4.70	<0.001
Head length [µm]	-0.09	0.07	47.63	-1.23	0.224
Head width [µm]	0.11	0.05	48.00	2.15	0.037
Head shape ratio	-0.10	0.03	47.97	-3.38	0.001
Head-flagellum ratio	<-0.01	<0.01	47.57	-3.28	0.002

Table S7 Effect of mean tail length on mean CASA velocity measurements using lm. 45 d.f.

CASA variable	Estimate	Std. error	*t*	p-value
Path velocity	1.72	1.33	1.29	0.204
Straight line velocity	2.91	1.29	2.26	0.029
Speed	1.08	1.47	0.73	0.466
Lateral amplitude	-0.08	0.08	-0.89	0.378
Beat frequency	-0.26	0.24	-1.07	0.290
Straightness	0.92	0.41	2.23	0.031
Linearity	1.03	0.44	2.32	0.025

Table S8 Effect of mean head width on mean CASA velocity measurements per sample using lm. 45 d.f.

CASA variable	Estimate	Std. error	*t*	p-value
Path velocity	17.73	18.34	0.97	0.339
Straight line velocity	29.51	18.01	1.64	0.108
Speed	-0.34	20.13	-0.02	0.986
Lateral amplitude	-1.10	1.16	-0.95	0.346
Beat frequency	-2.32	3.33	-0.69	0.490
Straightness	15.91	5.46	2.91	0.006
Linearity	17.54	5.87	2.99	0.004

Table S9 Effect of mean head shape ratio (head length:head width) on mean CASA velocity measurements per sample using lm. 45 d.f.

CASA variable	Estimate	Std. error	*t*	p-value
Path velocity	-37.57	28.80	-1.30	0.199
Straight line velocity	-56.51	28.12	-2.01	0.050
Speed	-10.41	31.84	-0.33	0.745
Lateral amplitude	2.02	1.83	1.10	0.276
Beat frequency	3.75	5.27	0.71	0.481
Straightness	-27.13	8.52	-3.18	0.003
Linearity	-28.71	9.23	-3.11	0.003

Table S10 Effect of mean head-tail ratio on mean CASA velocity measurements per sample using lm. 45 d.f.

CASA variable	Estimate	Std. error	*t*	p-value
Path velocity	-1250.43	1224.51	-1.02	0.313
Straight line velocity	-1937.11	1205.20	-1.61	0.115
Speed	-575.96	1343.06	-0.43	0.670
Lateral amplitude	74.96	77.58	0.97	0.339
Beat frequency	135.70	223.00	0.61	0.546
Straightness	-615.12	387.41	-1.59	0.119
Linearity	-652.34	418.32	-1.56	0.126

Table S11 Effect of acrosome reaction status on mean CASA velocity measurements per sample using lm. 41 d.f.

CASA variable	Estimate	Std. error	*t*	p-value
Path velocity	-37.50	46.77	-0.80	0.427
Straight line velocity	-28.89	48.77	-0.59	0.557
Speed	-87.36	48.72	-1.79	0.080
Lateral amplitude	-1.50	3.01	-0.50	0.620
Beat frequency	4.24	6.21	0.68	0.499
Straightness	3.25	7.17	0.45	0.652
Linearity	7.42	11.76	0.63	0.532

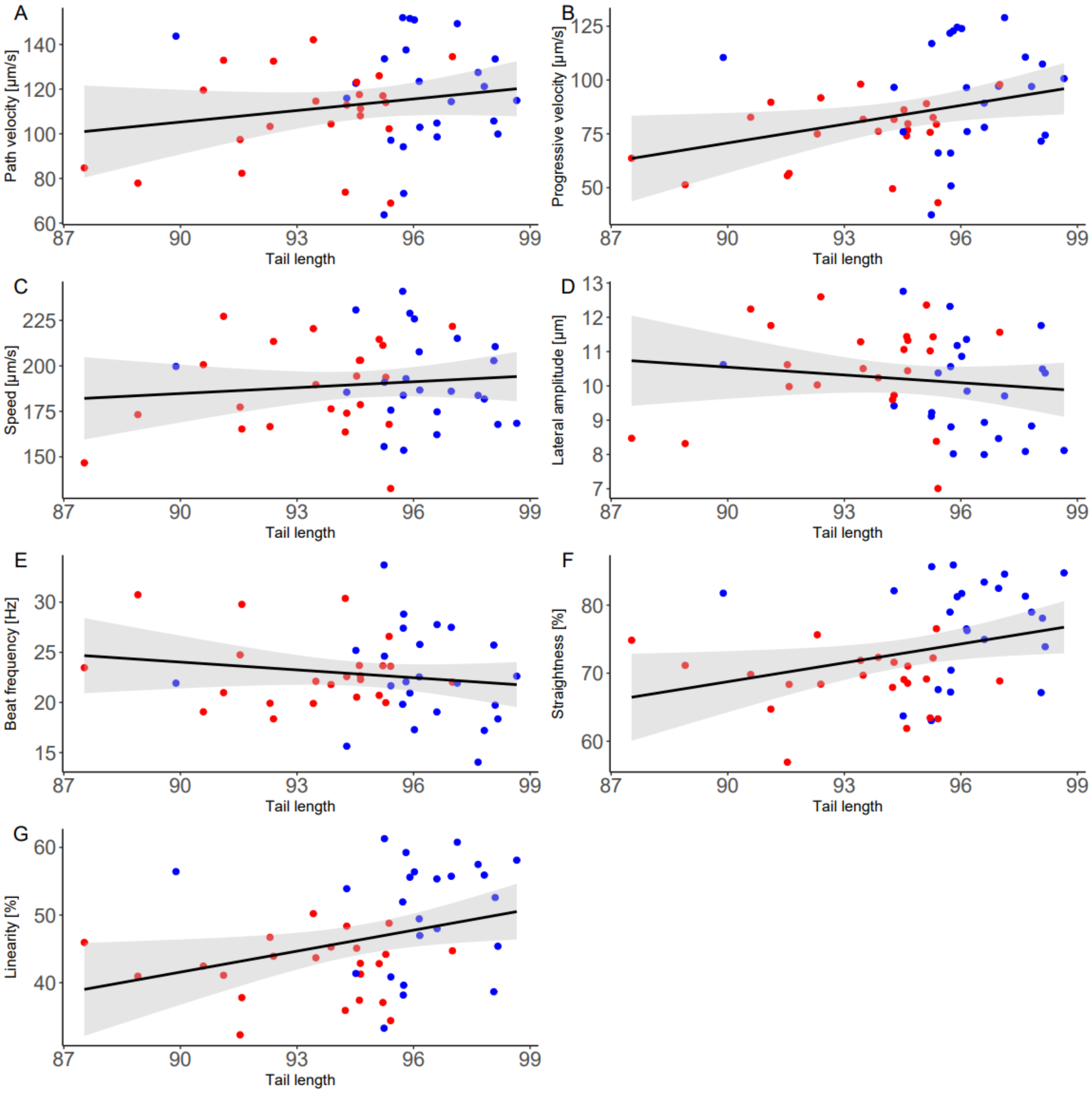

Fig. S1 Relationship between mean tail length and mean sperm motility for +/*t* (red) and +/+ males (blue).

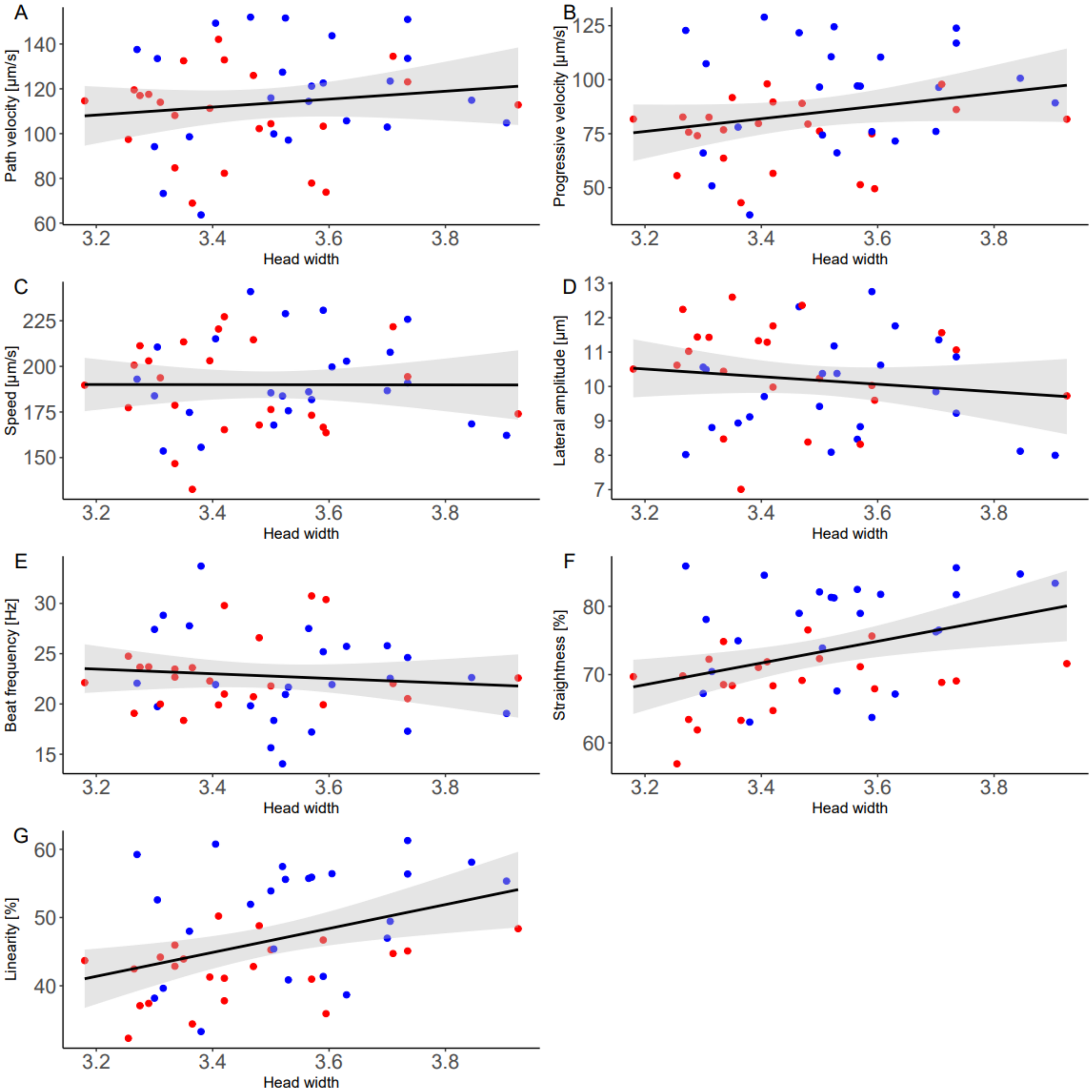

Fig. S2 Relationship between mean head width and mean sperm motility measures per individual +/*t* (red) and +/+ males (blue).

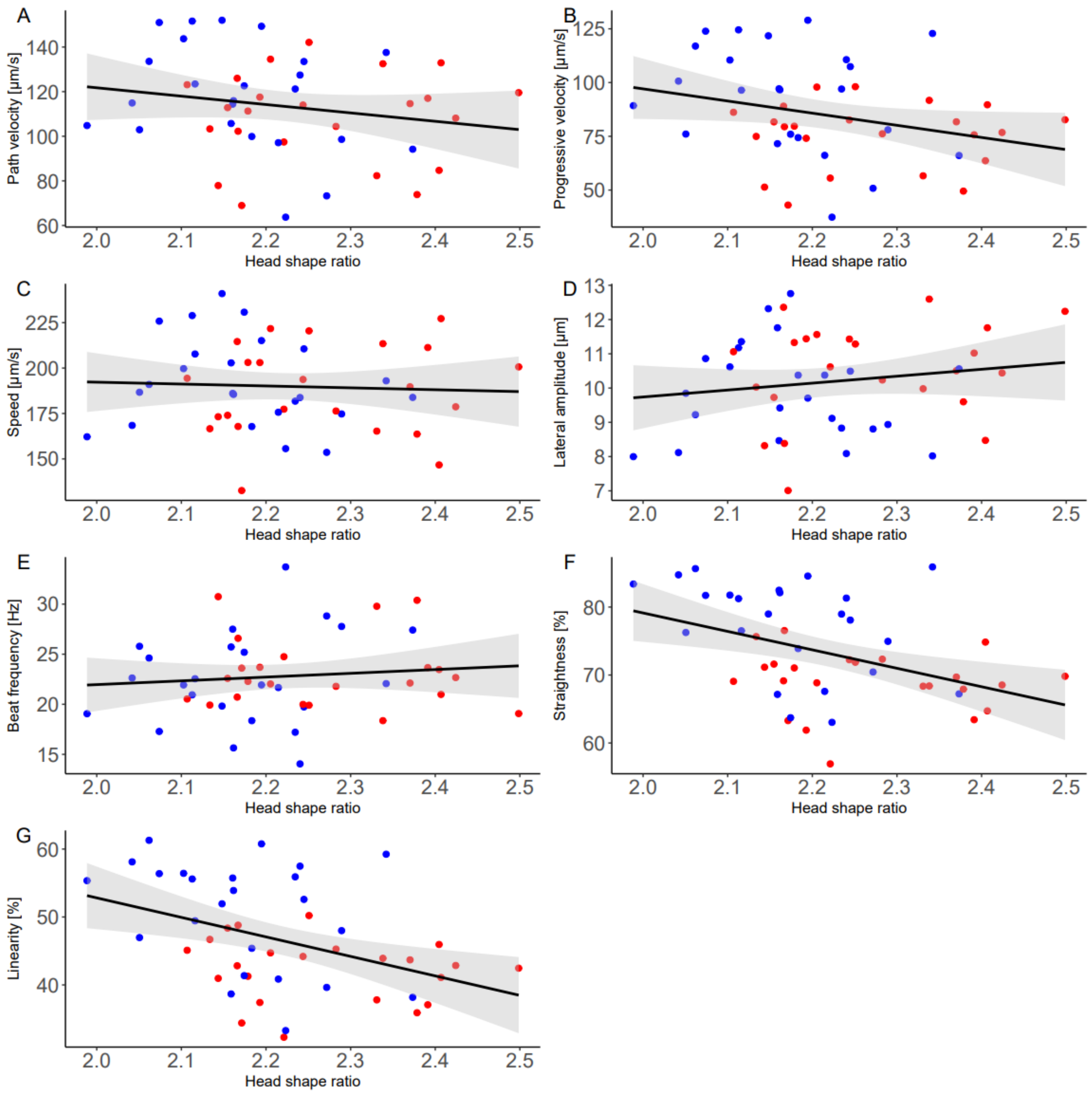

Fig. S3 Relationship between head shape ratio (head length:head width) and mean sperm motility measures per individual for +/*t* (red) and +/+ males (blue).

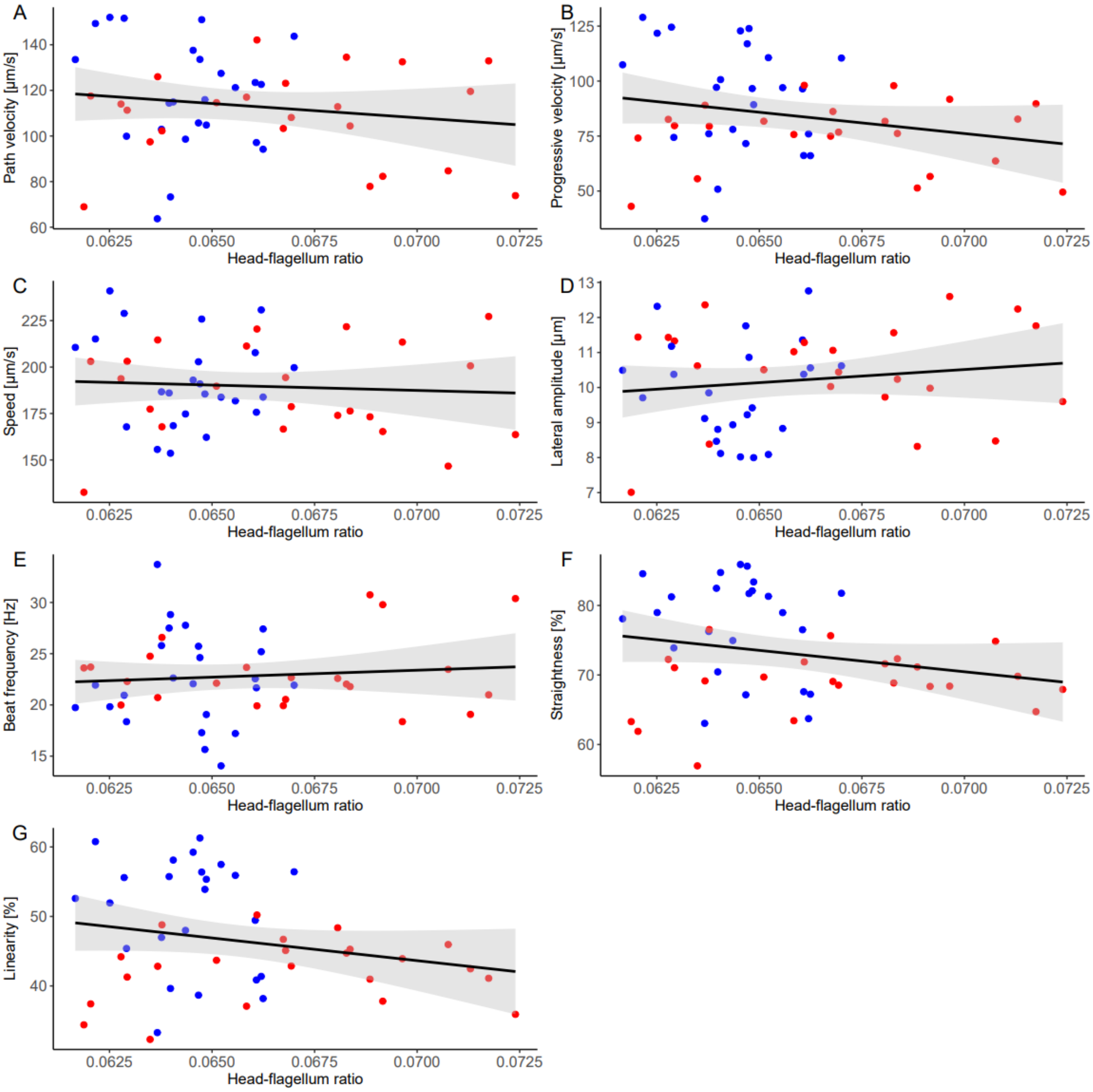

Fig. S4 Relationship between mean head-flagellum ratio and mean sperm motility measures per individual for +/*t* (red) and +/+ males (blue).

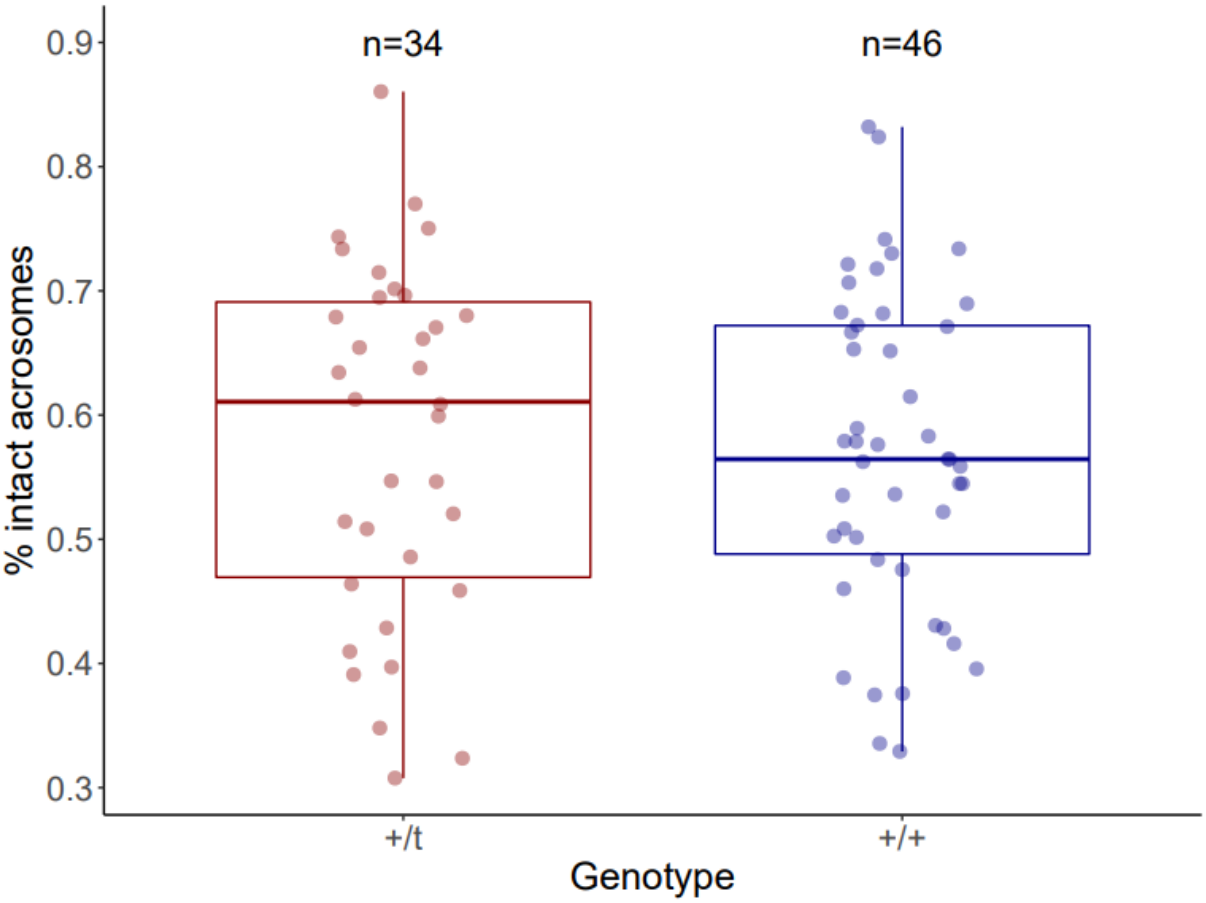

Fig. S5 Boxplot of +/*t* haplotype (red) and +/+ males (blue) by percentage of intact acrosomes. The midline represents the median and upper/lower limits the third/first quantile.

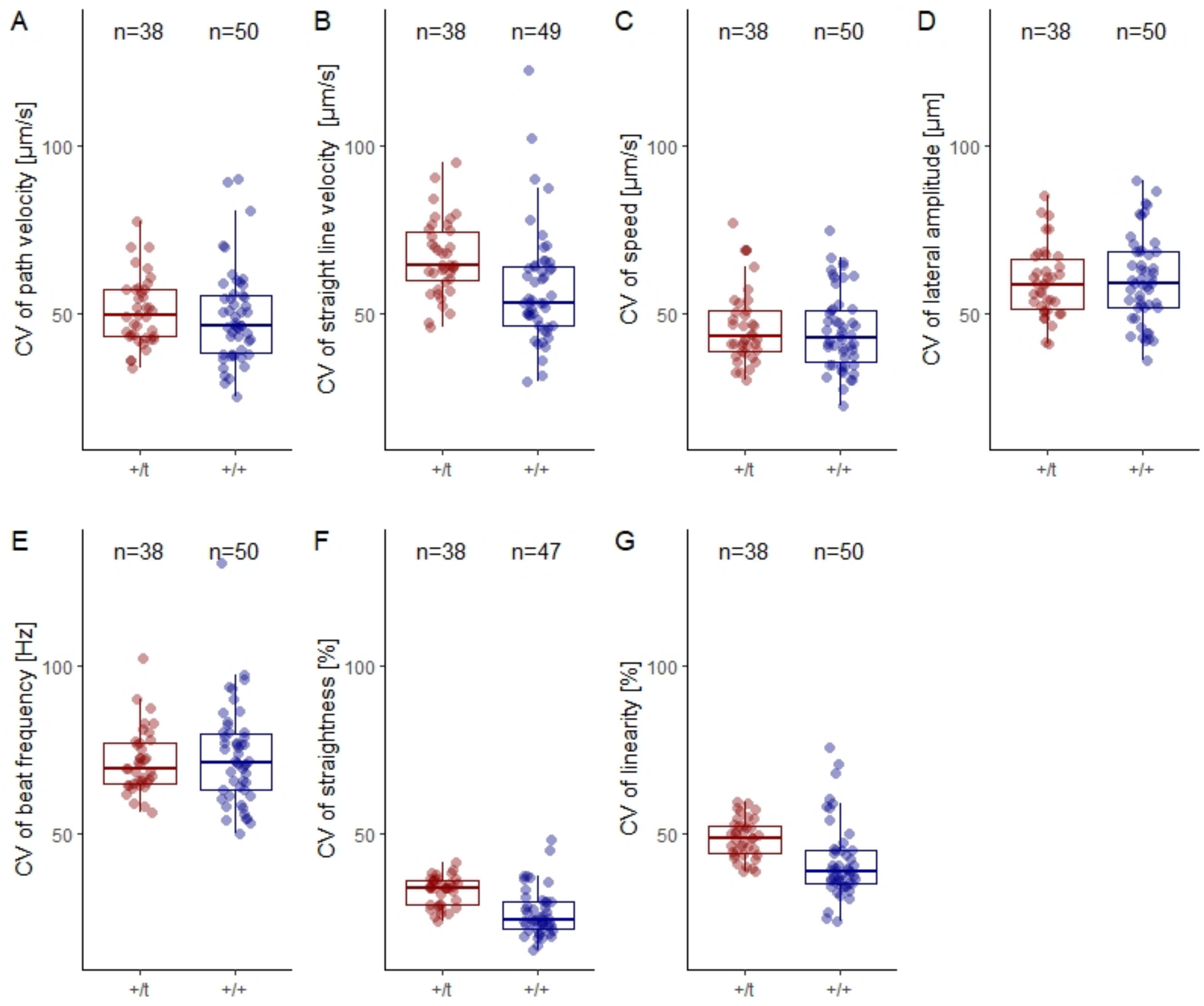

Fig. S6 Boxplot of coefficients of variation of sperm velocity parameters for +/+ (blue) and +/*t* males (red). The midline represents the median and upper/lower limits the third/first quantile.

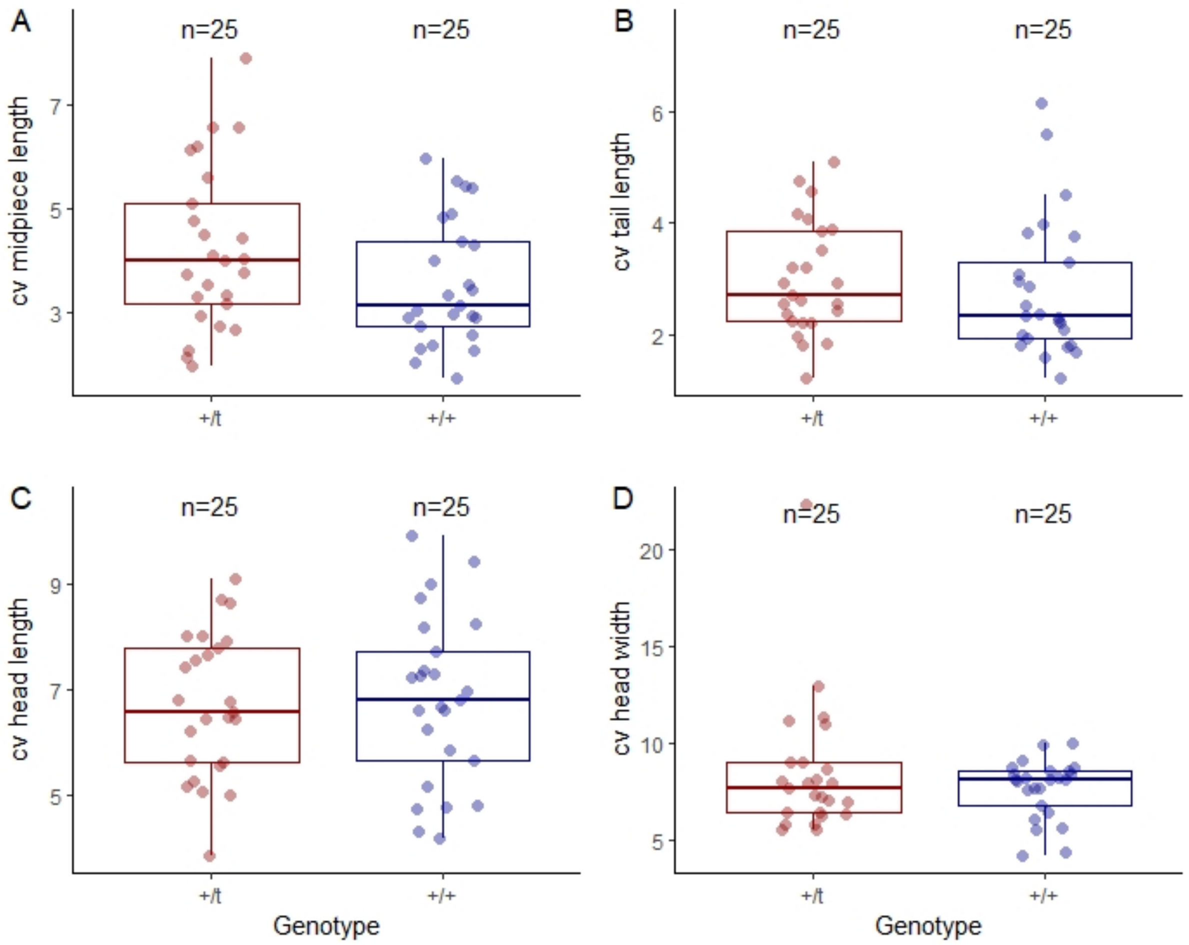

Fig. S7 Boxplot of coefficients of variation of sperm morphology parameters for +/+ (blue) and +/*t* males (red). The midline represents the median and upper/lower limits the third/first quantile.
